# Extension for Prevention

**Published:** 1901-03-15

**Authors:** C. M. Wright

**Affiliations:** Cincinnati, Ohio


DENTAL REGISTER.

Vol. LV. March 15, 1901. No. 3.

### EXTENSION FOR PREVENTION.

BY C. M. WRIGHT, D.D.S., M.D., CINCINNATI, OHIO.

Read before the Ohio State Dental Society, December, 1900.

When early in the summer I accepted the invitation of your committee to prepare a paper for this meeting of the Ohio State Dental Society, I exhibited a rashness of conclusion more becoming to a youth than to a conservative practitioner with a third of a century's experience, in giving as the subject of my paper the rather definite one, "Extension for Prevention." Your committee, having passed through an interesting and exciting political campaign since that time, juggled with my title, and it appears on your program to-day as "Expansion for Protection." Either will answer as a subject for the remarks I shall ask you to kindly consider at this time.

As I understand them, they both refer to certain operative procedures, recommended by distinguished dental teachers, in the methods of preparing cavities in the teeth for filling.

I have had an undefined opposition to these methods, which require an extensive cutting away of contiguous tooth tissues in the neighborhood of carious areas. The object of the cutting is to extend the borders between filling material and tooth, so that vulnerable lines and joints may be well exposed on labial and buccal as well as on lingual surfaces, and that cervical joints may be made under the free margin of the gum. The reasons for this being:

First. Because from recent microscopic examination it has been found that the dentin, as well as the bordering enamel, is often far more deeply and widely affected than ordinary chairside observations have been able to show.

Second. That the margins of cavities, simply filled, remain in the same distinctive environment, and recurrence of disease is liable at these places.

Third. That clinical observation has pointed to the history of operations. The small filling of gold or amalgam is often replaced in a few years by a larger, and this again in a few years more by a still larger, until the final filling has the dimensions recommended by this school as a first operation, by the extension of the original cavity walls.

Fourth. The prevalence and popularity of the doctrine, that by high-class and extensive operations we prevent recurrence of disease.

These are generally accepted as facts by most of us, and they may appeal to our judgment as reasonable grounds for the adoption of this extension for prevention, or protection, method of operating; and yet, as I remarked, I have been possessed by an undefined opposition or feeling of repugnance toward the method. We live in an operative age. The subdivision of medical practice into specialties has centered the attention of classes of medical men on special parts of the organism, and brilliant surgical operations have become the rule. It would almost seem, when we visit the consulting and operating rooms of the specialists of a great city, that man was placed on this earth to be operated upon. The dentist, and many special surgeons, seem to view him only as a subject. His nose, and throat, and mastoid region were given him that he might be operated upon. The vermiform appendix, the tonsil, the ovary, were developed in the human organism for the express purpose of being removed by a skillful surgeon. The more operations the surgeon performs the more skillful he becomes, and the happier man ought to be. The medical man who does not perform operations is not up-to-date and does not succeed in the professional race. There are reasonable grounds for these high-class operative procedures. We are not always in a position to dispute the doctrine which makes curetting a fashionable necessity this year, and yet, from the history of equally fine surgical measures, we may feel confident that it will not be resorted to next year.

It is not so very many years ago since the great operator, Arthur, offered a complete system for the surgical treatment of teeth by skillfully devised separations, for the prevention of caries. It was logical, reasonable and fascinating, and the dental profession, generally, accepted his teachings. Now it is a matter of history only, and has been entirely abandoned by the operators of to-day—yet who will say that, in the proper place and skillfully performed, it was not as successful as the author promised? To-day we protect with gold and restore contour, and the question has been boldly asked: Does filling save the teeth? The most favorable answer has been: "Yes, for a time, longer or shorter, depending upon certain conditions."

Twenty years ago the lamented Webb, whose memory we revere as one of the great lights of the operative world of the last half century, wrote a series of articles on "The Definite Principles of Filling Teeth." I must have felt the same opposition at that time that rises within me to-day, for I attacked these "Definite Principles" in an article entitled "Gold a Temporary Filling." Dr. Webb replied in a paper with the significant title "Gold a Temporary Filling, if Made Such," implying that gold properly applied and perfectly fashioned as a protective filling for carious teeth was or is a permanent operation. I have not seen these papers for many years, but the impression remains that Dr. Webb's definite principles would fit the "Extension for Prevention" doctrine of to-day; and, as well as I can judge, the method, or modification of it, forms the basis ot operative instruction in every first-class dental college of the present time. I freely admit this, and recognize that the proposition that the more perfect the filling—with all that this implies—the better the chances are for permanence, is like an axiom in our profession. Yet it is a fact that many imperfectly compacted and poorly finished and really inferior fillings, as far as mechanical skill and workmanship and material are displayed, seem to possess qualities of permanence and interference with recurrence of disease not possessed by neighboring and far more perfectly made operations.—*Kirk.*

Just here it may be well to recall another accepted article of our dental creed—one which does not hamper other operative specialists. It is the question of materials used in our surgery. When the term "permanent filling" is employed by dentist, dental teacher, or patient, the idea of gold first, and amalgam next, is presented to the mind. A permanent filling—or gold, or amalgam or some combination of metals, are interchangeable terms. A temporary filling means cement or gutta-percha. I have long regarded these terms, "permanent" and "temporary," as misleading and very serious obstacles in the way of scientific progress of our profession. Who first introduced the terms, or when in the dim ages of an ignorant past they entered the profession of dentistry, I do not know; but their universal establishment as recognized terms, applicable to the operations performed by the dental surgeon in his combat with disease, is certainly one of the distinctive misfortunes of our profession—a handicap in our race that has hindered the profession from arriving at the goal that so many of our early prophets and well-wishers have predicted and desired for us. I mean a high and secure standing among learned professions. If we could banish these words, and the ideas that the words wake up, from the minds of dentists and patients, we would make a step toward higher progress worthy of the twentieth century. The same terms, "Permanent and Temporary," operated to the disadvantage of the fathers of the profession in the department of prosthesis; so many of these early practitioners, in order to introduce themselves and their art, and to compete (possibly) with some brother dentist, were in the habit of extracting a lot of teeth for a patient, and making a temporary plate of silver for a small fee, or no fee, agreeing with the patient to make a permanent set on gold plate at the end of a year. The method was exceedingly demoralizing in its effect on the dentist—keeping him to the level of a tinsmith or jeweler; and to the patient, who was taught to dictate, and consider materials, instead of surgical and artistic service.

In operative dentistry, the same terms have marked us all, as with a stick of tar, and have kept us down, as a profession.

"I will fill this tooth with gold, for so much. This is a permanent filling," or, "I will fill it with a 'temporary filling.'"

Or, again, in a bill for professional services:

| | |
|---|---|
| Item A—One Gold Filling (meaning permanent) | $10 00 |
| Item B—Two Amalgam Fillings (meaning a cheaper kind of permanence) - - - - - | 5 00 |
| Item C—One Temporary Filling - - - | 1 50 |
| Why not ninety-nine cents? | |

The implication, the lesson taught the patient is, permanent and temporary treatment of caries, the temporary to be replaced by the permanent at some future time.

My personal repugnance to the ideas which I have tried to make clear, in all fairness, in these pictures, may have accounted for "undefined" objections which I felt for the "extension for prevention" methods of operating. It smacks of the permanent versus the temporary; while all our teaching should be, that the cause of the disease is, in most cases, the only permanent thing in the case. About twenty years ago a dealer in dental goods from Berlin said to me, "What do you think of the honesty of a dentist who uses pink gutta-percha for filling teeth? The plate gutta-percha I mean, at five marks a box. Isn't that pretty low down?" Just before that, all unconscious of the emphatic scorn of a dealer for a dishonest dentist who would employ a cheap laboratory material for filling teeth, I had innocently and publicly made the statement that I believed that a dentist could take care of the teeth of 2,500 persons of all ages each year, with the minimum amount of pain and the maximum amount of comfort, using only pink gutta-percha as a filling material—provided, that he could have the intelligent co-operation of the 2,500 patients themselves. This co-operation implying an acceptance of the fact that treatment was temporary, and the disease chronic ; that prevention depended upon a removal of the cause ; that fillings do not affect the cause.

This was a bold statement, full of dental heresy—if I may use this expression—for a young man to make.

After two decades of continuous practice, I still entertain the same heretical opinion—though I have never been permitted, for various reasons, such as fear of public opinion, the habit of practicing in a rut, and lack of courage to face possible consequences to my purse—to prove my theory. The idea here expressed is the reverse of that of the method of "Extension for Prevention," because the "permanent" is banished and the temporary, simple and painless repair of waste tissue rises from the horizon like the morning sun, shedding beneficent rays of promise to all the world. The motto of an army of dentists practicing on these saving lines would be, "Eternal vigilance is the price of—good teeth."

The more wearing and painful and extensive and difficult the capital operation on one tooth, with the possibility that it may last ten years, instead of the easy, the painless, the delicate and the simple operations on ten teeth that may possibly (nay, probably) require the same treatment in two or three or five years, seems to be the fixed authoritative method of thought and action and teaching of to-day for first-class dentistry ; and gold—until we begin to consider cosmetic and esthetic qualities—has been the only high-class material for these orthodox operations. One of the prominent arguments which holds gold as the first among so-called permanent materials, is, strange to repeat, because of the difficulty in applying it to positions in living teeth, or teeth in a living mouth—this very difficulty tending to increase the manipulative ability of the operator by reflex action, and co-ordinated with this is the secondary reflex good to the patient, who gets the benefit ot the very skill his or her endurance and suffering has helped to establish. What would we think of a surgeon in other fields who would employ only gold wire for ligatures, because the difficulty of its employment added to his skill, when he might employ silk or catgut, which are easy to tie?

Many years ago the elder Coffin, of London, abandoned all materials for filling teeth, excepting only chlorid of zinc. This attitude toward the profession and his exclusive and titled patients, on the part of a cultivated and distinguished dentist, made a deep impression on my mind. It seemed then, as it does now, that the move was in the right direction, and away from the hampering and demoralizing notion of permanency and the narrowing orthodoxy that makes the ability to manipulate gold foil the standard of perfection in the dentist.

The prize fillings of the graduating class of a dental college are finer mechanical specimens than the fillings made in daily practice by the experienced and often distinguished examiners, selected as they are from among the best urban operators. The new graduate, then, is a better dentist than he will be in ten, twenty or thirty years of practice. The older man can accomplish these same fine mechanical results, built up as they are on definite principles, but he gradually ceases to do so. Why? Is it because, while still maintaining the orthodox in theory, he becomes emancipated in practice, and leans toward the heresy which I have tried to portray, and to perhaps establish as sound doctrine?

A couple of years ago I read a short paper before the Odontological Society of Cincinnati, and proposed the following question: Suppose that, for any reason, you were compelled to select but one material for filling teeth—all teeth—which of the now known materials would you adopt? There were present some twenty members, who kindly gave the question their earnest attention and each man present answered for himself, and according to his judgment. Not one selected gold as the filling material that he could depend upon for "saving" all teeth. Finally, gutta-percha and the cements—the majority selecting gutta-percha, and the rest cement—temporary fillings were selected as the real sheet anchors of the dental bark in the harbor of salvation.

An incident of interest during the discussion was the astonishment of the younger members, some of whom had had but a few years' practice out of college, at the opinions expressed by the experienced dentists of that society. "It is so contrary to what we have been taught;" but was it not food for thought, or seed sown, that may spring up in due time and bring forth sounder professional doctrines for the future?

If the members of this State Society will lay aside for a few moments, all prejudice, and wipe out tradition with its potent influence on our minds, and consider *de novo*, each one for himself, his position toward this question, there may be a refreshing revival of reason—the scales may fall from our eyes, and we may see men, as trees, walking, as did the blind man whom the Savior cured—or in other words, we may begin as little children to see things for the first time, and form sounder judgments than have been possible with the scales of authority, of teaching, of proverbs and of ancient laws, before our eyes. Let me place before the new eyes of thought these propositions:

Gold is a filling material, like lead or tin, for the temporary repair of tooth lesions.

A man may be a fine operator and an honest man, and employ only cement and gutta-percha in his practice, and charge good fees for his services.

It seems to me to-day, as it did years ago, that if we could, as a profession—as teachers and practitioners—see things from this standpoint, we could advance dentistry as a science and as an art, because of the more rational and scientific groundwork upon which the whole structure would rest. Then, the physical exhaustion from one form of manipulation would be lessened and we could bring less tired nervous energy to bear on broad questions of importance, as for instance, on diathesis.

Suppose that we could establish as a fact, the proposition of some late medical investigators, made independently of the views first enunciated and published by Dr. Paul Gibier, on the influence in pathology of the prevalence of the alkaline, or, of the acid temperaments and dispositions. Suppose that we could say, from a chemical standpoint, that all men may be divided into three classes, viz.: the acid, the alkaline and the neutral, and that the first two comprise the majority of the sick, whose body fluids appear to contain an excess of acids, as in the rheumatic and the gouty, or an excess of alkali, as in the strumous and tubercular; that the neutral, or those in whom there is a fair and proper balance between acidity and alkalinity, are apt to be the healthy people. What a field of study would be opened to us in the line of dietetic and systemic treatment in our specialty that we might be able to combat a predisposition to excess in either direction, in order to maintain the neutrality, which, if we could establish the fact, alone seems compatible with prolonged good health and tissue preservation, whether that tissue be tooth, or gingival, epithelial or connective, vascular or nervous.

The idea of "permanency" then could be regarded as depending more upon the body fluids than upon extensive excavations with engines and tools. We could then announce authoritatively once for all, that fillings are not made as preventive measures; that other than operative procedures must be employed for prevention, and that these must be found in constitutional or systemic therapeutics.

The efforts of the dentinal pulp in protecting itself from even minor traumatic or chemical irritations should remind us that even a small cavity of decay in a remote corner of a tooth becomes, in a sense, more than a mere local disease—for the organism is called upon to protect and repair, by modified cicatricial tissue in pulp and dentinal tubules, an injury to the periphery.

Have I said enough to make the picture that I have in my mind's eye clear to you, and to offer reasons why I shrank from the "extension for prevention" proposition, brilliant and apparently reasonable as this method appears from the standpoint of operative therapeutics?

### DISCUSSION.

Dr. F. A. Hunter: In regard to the "Extension for Prevention," so-called, it is a very pretty theory, which is practical in some few instances, and if the advocates of that theory will say it is applicable occasionally I will agree with them; but when they lay it down as a rule it seems to me in the rough and tumble practice of daily life, when we are working on living patients, our ideals can not be carried out. Although practiced to a great extent by all of us, it must be governed by conditions, to lay it down as a rule I feel the same as Dr. Wright, it is carrying the thing too far. There are very few men in the profession who have the ability to perform these operations, even, as I might say, on a dead subject, much less on a nervous, irritable patient, who is alive to all the feelings we have. To enlarge upon the proximate surface of a small cavity with well defined walls, for the purpose of having self-cleansing, is, in my mind, carrying the matter entirely too far.

Dr. Stephan: We should not forget that it is our life's work to extend for prevention. In every operation we perform we extend to prevent the inroads of caries or of disease. We cut away the diseased portions of the tooth substance and replace the lost parts of the tooth in order to prevent it from a more destructive course. I do not take it that the doctrine of extension for prevention is to be followed in the working of metallic fillings alone; it should be followed in the use of plastics or temporary fillings as well.

Extension for prevention does not necessarily mean cutting away a great amount of tooth substance in every case; judgment is to be used, as in everything else. The writer has carried his idea to the cutting of the bicuspids and molars and in anterior teeth so that the labial, lingual and buccal walls or margins will be kept clean by the cheek and tongue. This doctrine also goes beyond cavity extension. We should restore with the filling the tooth to its original shape. Many operators fail to consider the question of rebuilding the natural contour of the tooth. The closer we cling to natural conditions the better our results will be.

I thank Dr. Wright for this most excellent paper, also Dr. Johnson for his paper. One extreme in each direction will give us food for thought.

Dr. H. H. Harrison: The old colored adage says: "Keep in the middle of the road." That is just what I think we should do here. I have enjoyed the papers, and yet there is that feature of the extreme in either direction that is dangerous. According to my idea the preparation of any cavity is simply to take away all the decomposed elements of the tooth structure, all partially degenerated tooth substance, and form so as to build in whatever kind of a filling. By simply taking away all that is absolutely decayed, and thoroughly sterilizing the remainder, you have done all that is necessary before filling the cavity.

Dr. C. R. Butler: I do not exactly comprehend the position that Dr. Wright has taken in the presentation of this subject. It seems he has not taken the extreme either way, but simply emphasized the mistakes that had been made in some of our teachings and nomenclature. It seems that the benefit we are able to extend or bestow upon our patients, or the lack of it, is more within our grasp than perhaps we are disposed to think. Men who have been in practice as long as Dr. Wright, and others here present, have learned that by some indescribable influence, when we do for our patients what we please, they are conscious of whether we are doing our best or simply compromising. I

apprehend that there has been too much promised on the part of the profession. The idea of saying, "Now, that tooth is filled forever; it will last you your lifetime," etc. I should modify that by saying, "if you don't live too long."

Some here have got the idea that Dr. Wright is attempting to make an explanation for the use of extension for prevention. But Dr. Wright does not employ that principle in his practice; he has seen the mistake of such practice and has heard so much of it for some time past that he really does not know what is best. The cutting away of good, sound tooth substance, to be replaced with a non-vital substance, he does not regard as prevention. He does believe, however, in taking away all the diseased portion of the tooth and in trying to put a substitute there which will save the organ as long as possible. That is what we should all do and use less frequently the terms permanent and temporary. Patients in the old country get the idea that dentists are serving them temporarily; they expect to go and have their teeth stopped once a year. But we Americans try to do a little better than that; we try to put in a filling which will last for several years; this is simply a modification of the permanent idea. To say simply, "It may last a lifetime," is sufficient. What concerns us principally as dentists is to see how well we can serve those coming to us needing our services so they may be relieved of pain and discomfort, and have the teeth with which nature has provided them perform their functions in the best possible manner.

Dr. Wright (closes discussion on his paper): Mr. President: I have had the advantage of Dr. Johnson to-day; I heard his paper, he did not hear mine. I do not know that I have anything special to say in closing the subject. As far as my impression of Dr. Johnson's paper is concerned, I thought it was a most vivid description of a magnificent operation. It is to such men that our beloved profession owes its distinction, and it is to our operative skill that the American dentist is known the world over. While I am willing to admit that the very distinction, which we as a profession have attained, is largely due (I won't say solely due) to our technical skill, our manipulative perfection; and, while I consider digital expertness a *sine qua non* of the dentist, I still have deep regrets that our fundamental doctrines are unsound; that the reasons we give for our fine operations are not true, and it is for this reason I raised my voice this morning against this blot on our otherwise spotless escutcheon. I do not stand alone in this position. Dr. T. C. Stockwell, of Springfield, Mass., lately read a paper before the Massachusetts State Dental Society which was published in the October, 1900, *Cosmos*, on "Prenatal Treatment of Teeth," in which in no uncertain sound he warns our profession against the unprofessional satisfaction it takes in fascinating operative procedures to the possible neglect of higher scientific culture. Dr. Kirk, in an editorial a month or two ago, strikes the same chord in his significantly earnest way. Every now and then we have these sentiments coming as warnings from thinking men, not from cranks. It is probable, however, that the majority of the profession are inclined to follow the glittering operations.

---

To REMOVE the offensive smell of iodoform, wash the hands in soap and water, rinse with dilute aqua ammonia, after which apply lemon juice or vinegar.—*Eclectic Medical Journal*. We find Johnston's Ethereal Antiseptic Soap very efficient for this purpose.—ED.

